# A hospital based epidemiological study of snakebite in Paschim Medinipur district, West Bengal, India

**DOI:** 10.1016/j.toxrep.2017.07.007

**Published:** 2017-07-24

**Authors:** Sumana Sarkhel, Rituparna Ghosh, Koushik Mana, Kripasindhu Gantait

**Affiliations:** a Department of Human Physiology with Community Health, Vidyasagar University, Paschim Medinipur-721102, West Bengal, India; b Department of Medicine, Midnapur Medical College, Paschim Medinipur-721102, West Bengal, India

**Keywords:** RH, rural hospital, BPHC, Block Level Primary Health Centre, CMOH, Chief Medical Officer of Health, PHC, Primary Health Centre, ASV, antisnake venom, BMC, Burdwan Medical College, AchEIs, acetylcholinesterase inhibitors, Snakebite, hospital -based data, Paschim Medinipur

## Abstract

**Objective:**

Snakebite is a serious medical problem in *Paschim Medinipur* district, West Bengal, India. In the present study, hospital based data on snakebite cases and deaths were collected from 10 blocks of the district to obtain a picture of this neglected public health issue.

**Methods:**

Retrospective data of snakebite and deaths from 2012 to 2016 was collected from the 5 Rural hospitals (RH) and 5 Block level Primary Health Centres(BPHC) of the ten blocks of *Paschim Medinipur* district in a prescribed format addressing issues including the demographic factors regarding the victims, seasonal pattern, history of snakebite in previous 5 years, outcome of the bite, any reporting of death.

**Results:**

A total number of 1633 snakebites were reported in the study during the period of 2012–2016. The hospital data from the ten blocks reported 17 deaths due to snakebite in the given period. It was found that about 34% of the snake bites occurred from June to September. The age-wise distribution of snakebite cases show that majority of snakebite affected cases were within the age of 21–45 years. Males(62%) suffered more than the females(38%). The case fatality rate reported from the hospital based data was 1.04%. 60% of the snakebites occurred during morning to noon hours. The clinical manifestation of snakebite included regional edema in the affected limb, hypotension, malaise, vomiting, abdominal pain and diarrhea. The bite to hospital time was found to be 120 ± 6.5 mins(n = 750 cases) and bite to ASV injection time was found to be 270 ± 3.5 mins(n = 750 cases).

**Conclusion:**

Snakebite is a neglected health issue in *Paschim Medinipur* district. Lack of snakebite data is due to under reporting. In the present study we have attempted to investigate the official data available on snakebite from 10 blocks of *Paschim Medinipur* district which has been identified for high snakebite incidence.

## Highlights

• The present study highlights the medical problem of snakebite in Paschim Medinipur district, West Bengal.
• The case fatality rate of snakebite from 10 block level hospitals have been reported in the study.
• The symptoms and management of snakebite has been discussed.

## Graphical abstract

**Figure:**
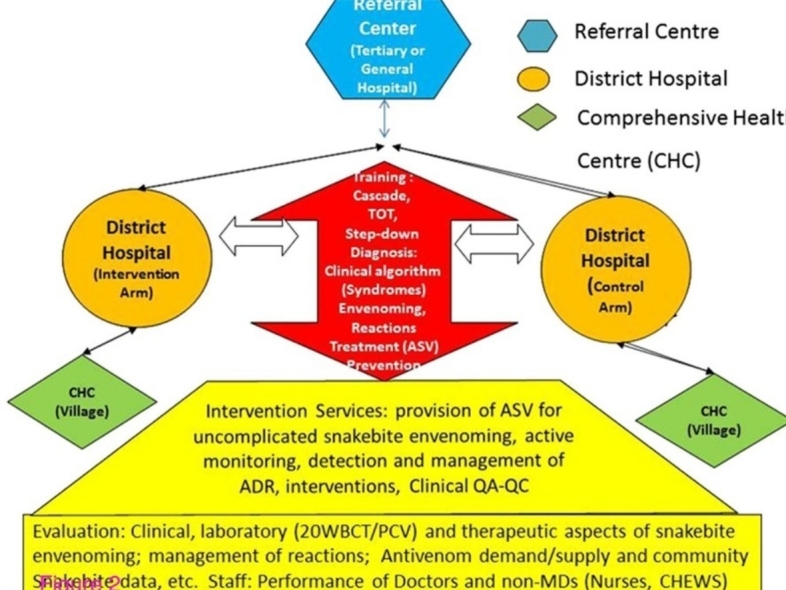

## 1 Introduction

Snake bite was included in the list of neglected tropical diseases by World Health Organization in the year 2009 [1], [2]. Globally every year, an estimated more than 5 million people are bitten by snakes, [3], [4] resulting in approximately 20,000–1, 25,000 deaths [5]. West Bengal is one of the high snake bite prevalence states of India besides Andhra Pradesh, Kerala, Tamil Nadu and Maharashtra [6], [7], [8]. Rural West Bengal in India showed an average annual mortality rate of 16 per 100,000 population [6].

Accurate data are often difficult to find as bites affect rural populations in remote areas with limited access to formal health care [9]. There are very few community-based surveys on mortality related to snakebite, and these have shown high rates. Many estimates of snakebite incidence and mortality are based on hospital data, because other recording systems are unavailable or unreliable in most developing countries. However, hospital data may considerably underestimate the problem. In Africa, it is estimated that less than half the deaths due to snakebite are reported by the health services, and similar under-reporting is likely to occur in most African and Asian countries where snakebite is prevalent [10]. In remote rural areas of the tropics it is estimated that a third to more than half of snakebite victims do not receive treatment at a hospital. Thus, a proportion of snakebite deaths can occur before the victims can reach a hospital, and in some instances victims with fatal bites may not attempt to use formal health care services [11].

## 2 Methods & materials

To investigate the snakebite scenario of *Paschim Medinipur* district, West Bengal we have randomly selected 10 blocks (out of 29 blocks) for this study. The blocks are- **Pingla, Chandrakona I, Chandrakona II, Debra, Kharagpur I, Kharagpur II, Salboni, Keshiary, Garbetta I** and **Garbetta III.** This study was financially supported by Department of Science and Technology, West Bengal, India. There are 30 Primary Health Centres(PHC), 5 Block Primary Health Centres(BPHC) and 5 Rural hospitals(RH) in these ten blocks (Table 3). Prior permission was obtained from Chief Medical Officer of Health (CMOH) of *Paschim Medinipur* for this study. The snakebite data over past five years were collected from the BPHCs and PHCs. The information regarding seasonal pattern of snakebites, number of patients admitted and the number of deaths due to snakebite from 2012 to 2016 were collected from the databases of health centres of the ten blocks. The demographic information about the population, male: female ratio, percentage of rural and urban population of the study area was collected from District Magistrate’s office of *Paschim Medinipur* district.

The information collected from the Chief Medical Officer of *Paschim Medinipur* district included- names of the government health facilities (BPHC or Rural Hospitals) in the blocks, number of snake bite cases who had attended the government hospitals in the blocks under study, number of snake bite deaths reported from the study area.

Evidence of bite by a poisonous snake included: (i) fang marks, (ii) swelling, ecchymosis, blister formation and/or bleeding from local site, (iii) disturbances in coagulation mechanism with or without systemic bleeding, and (iv) identification of snake wherever possible (Table 1). Swelling confined only to the bite site was graded as mild; extension to more than half of the limb as moderate; and extensive swelling with tissue necrosis was graded severe [12], [13]. Neurotoxicity was defined as documented ptosis, external ophthalmoplegia, weakness of neck or bulbar muscles, use of neostigmine or ventilatory support (endotracheal intubation or a mechanical ventilator). The clinical gradation of snakebites and envenomations is crucial for evaluating and managing the condition of each victim [14]. According to the patient condition at the time of admission, it was classified as follows [15] (Table 2):

**Table 1:** Types of Envenomations.

Type of bite	No of cases reported (n = 950)(%)
*Vipera russelli (Chandrabora)*	570(60%)
*Common Krait*	238(25%)
*Naja Kaouthia* (Keute)	143(15%)

n- Number of cases where the type of snake was identified.

**Table 2:** Clinical grades of envenomation.

Grades of Envenomation	Number of cases(n = 820cases)(%)
Grade 0	51(6.2%)
Grade 1	250(30.5%)
Grade 2	335(40.8%)
Grade 3	159(19.4%)
Grade 4	25(3%)

**Grade 0**: bite without envenomation (absence of edema or local reaction).

**Grade 1:** minor envenomation (local edema, absence of general signs).

**Grade 2:** moderate envenomation (regional edema in the affected limb and/or moderate general symptoms: moderate hypotension, malaise, vomiting, abdominal pain and diarrhea).

**Grade 3:** severe envenomation (extensive edema reaching the torso and/or severe general symptoms: prolonged hypotension, shock, anaphylactoid reaction and visceral trouble).

**Grade 4:** extremely severe envenomation (apparent systemic involvement with blood-tinged secretions, renal failure, coma and death).

Analysis was carried out using Excel^®^ (Microsoft, USA). The **case fatality rate** of the hospital data were calculated by the following formulas-

**Case fatality risk**, case fatality ratio or just fatality rate — is the proportion of deaths within a designated population of “cases” (people with a medical condition), over the course of the disease.

fatality rate = Notified deaths × 100/Notified cases = ɑ deaths/100 cases

## 3 Results

**Fig. 1:**
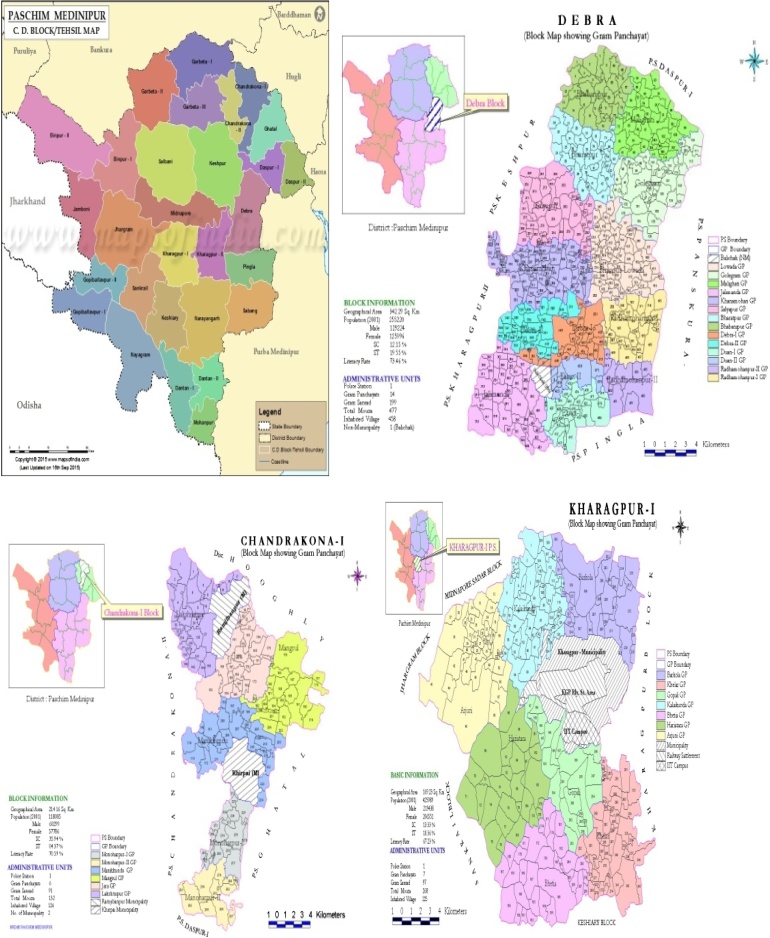
Map of the study area covering blocks of Debra, Chandrakona- I, Kharagpur I.

The study was conducted on a population of 2145572(32.5% of total population) from 10 blocks of *Paschim Medinipur* district(Fig. 1). The total population of the district is 6598469. The male to female ratio of these blocks is **1.03**. From the survey the total number of snakebite cases reported from the block level health centres and rural hospitals were **1633**. Of the total number of snakebite cases **1006(62%)** were male and **627(38%)** were female. A total of **17 deaths** were reported from **5 years** hospital based retrospective data of 10 blocks between 2012 and 2016 (Table 4). The case fatality rate reported from the hospital based data was **1.04%.** Maximum(**34%)** snakebite cases were reported in June to September months. Most of the snakebites occurred in the rural areas during agricultural activities. The agewise distribution of snakebite cases show that majority of snakebite affected cases were within the age of 21–45 years. However due to lack of proper documentation in 850 cases the age was not mentioned in the report (Table 5). The **bite to hospital time** was found to be **120± 6.5 mins(n** **=** **750 cases)** and **bite to ASV injection time** was found to be **270** **±** **3.5 mins**(n = 750 cases). In **60% (n** **=** **950 cases)** the snakebite occurred between the early morning hours and 4pm.

**Table 3:** List of BPHC, PHC and RH in 10 blocks of Paschim Medinipur district West Bengal.

Block	Name of BPHC & RH	Name of PHC
Garhbeta I	Garhbeta RH	• Sandhipur PHC
		• Parbatipur PHC
		• Nohari PHC
Garhbeta III	Dwarigeria BPHC	• Nayabasat PHC
		• Chototara PHC
Chandrakona I	Khirpai BPHC	• Ramjibanpur PHC
		• Ramkrishnapur PHC
		• Mangural PHC
		• Dingal PHC
		• Jara PHC
Chandrakona II	Chandrakona RH	• Basanchora PHC
		• Bhagabantapur PHC
Salboni	Salboni RH	• Godapiyasal PHC
		• Bhimpur PHC
		• Pirakata PHC
Kharagpur I	Hijli RH	• Khelar PHC
		• Khemasuli PHC
		• Amba PHC
Kharagpur II	Changual BPHC	• Gokulpur PHC
		• Paparara PHC
Debra	Debra RH	• Satyapur PHC
		• Pasang PHC
		• Trilochanpur PHC
		• Kakra Shibrampur PHC
Keshiary	Keshiary BPHC	• Patharhuri PHC
		• Ganasarisha PHC
		• Khajrabari PHC
Pingla	Pingla BPHC	• Harma PHC
		• Jalchak PHC
		• Boalia PHC

**Table 4:** Blockwise incidence rate of snakebite of Paschim Medinipur district (from official data of 2012–2016).

Names of the blocks	Population in the block	Reported hospital attendance		Reported hospital deaths (5yrs)	Case fatality rate(of 5yrs)
		Male	Female		
Garhbeta I	256589	312	207	06	1.16
Garhbetta III	190743	117	64	–	NA
Chandrakona I	151083	30	20	–	NA
Chandrakona II	138500	152	100	09	3.5714
Salboni	211723	106	53	–	NA
Kharagpur I	286717	4	1	–	NA
Kharagpur II	205967	3	0	–	NA
Debra	320478	236	151	02	0.5167
Keshiary	166244	30	27	–	NA
Pingla	217528	15	4	–	NA
Total		1005	627	17	

NA: Not applicable.

**Table 5:** Agewise distribution of snake bite cases.

Age-wise distribution (Years)	Male	Female	Total
0–10	43	16	59
11–20	80	51	131
21–30	97	70	167
31–40	93	67	160
41–50	97	50	147
51–60	77	20	97
61–70	21	05	26
70+	08	02	10
Age not provided	512	338	850

### 3.1 Signs & symptoms of envenomation

Local pain at the site of bite was reported by 100% of cases admitted to the block level hospitals. Edema and coagulopathies were reported by 335(40%) along with moderate hypotension, malaise, vomiting, abdominal pain and diarrhea. Severe envenomations was characterized by prolonged hypotension, shock, anaphylactoid reaction and even death.

### 3.2 Treatment & follow-up

All patients received tetanus toxoid and antisnake venom (ASV).The patients were treated with polyvalent antisnake venom and most of them received single doses (10vials) and in severe envenomations single doses(10 vials) were repeated upto three hours. However about 20% of the cases developed anaphylactic and pyrogenic reactions. The pyrogenic response was treated with paracetamol and the anaphylactic response was managed by adrenaline(*i.m*), hydrocortisone(*i.v*) and ranitidine(*i.v*). The mean bleeding time was 10.55 ± 3.2 min(n = 862 cases). The mean clotting time was found to be 16.1 ± 2.55 min(n = 862 cases). Neostigmine was provided to 80(35%) of the patients who developed signs of neuroparalysis. The mean duration of stay of all patients at the hospital was 8.5 ± 2.2 days(n = 950 cases). Ventilatory support was provided to 30 patients.

## 4 Discussion

West Bengal is one of the high snake bite prevalence states of India besides Andhra Pradesh, Kerala, Tamil Nadu and Maharashtra^8^. Government of India official data showed only 1331 snake bite deaths in the year 2007. If compared with another highly attended public health problem, malaria, we see officially reported numbers of snake bite deaths are much higher than malaria death in West Bengal (340 and 96 in 2007 respectively**)** [16]. Epidemiological surveys on snake bite in India are primarily based on hospital records [6]. There is no snake bite data of *Paschim Medinipur* district for last 15 years that is based on demographic surveys and retrospective health centre surveys.

*Paschim Medinipur* is one of the eleven districts of West Bengal. Geographically located in 21°36′ to 22°57′North latitude to 86°33′ to 88°11′ East longitude, it covers an area of 9345 square kilometres. North and North west of this district is a part of Chotanagpur plateau. The area has a gentle slope from east to west. The sandy loam or loamy soil of reddish or reddish brown colour is a characteristics of this region. The maximum temperature recorded in April is 45°–46 °C and minimum temperature in winter is 6 °C. The average annual rainfall is about 1500 mm. *Paschim Medinipur* is diverse in geography, climate, flora and faunal resources. The tribal communities residing in this region are the *Santhals*, *Mundas, Lodhas*, *Bhumijs, Oraon* and *Kherias*.

Early initiation of treatment by anti-snake venom (ASV) serum is the key of low case fatality rate [17]. In the present study, the bite to hospital time was found to be 120 ± 6.5 mins(n = 750 cases) and bite to ASV injection time was found to be 270 ± 3.5 mins(n = 750 cases).In the present study the patients were treated with polyvalent antisnake venom and most of them received single doses (10vials) and in severe envenomations single doses(10 vials) were repeated upto three hours. A similar study, accomplished in northern Bangladesh, registered seven deaths (26%) among victims treated with anti-snake venom doses ranging from 20 ml to 40 ml [18]. A prospective study of hospital practice in the Gampaha district, Sri Lanka, documented only 0.43% mortality when at least ten vials (100 ml) of antivenom were initially given to victims [19]. These findings agree with Theakston et al., [20], who concluded that is prudent to give initial doses greater than 100 ml to patients with neurotoxic signs, as well as is necessary to repeat this dose every few hours, up to a total of at least 300 ml, if there is no response.

In this study the case fatality rate from the official hospital data was found to be 1.04%. Case fatality rate of a tertiary referral hospital of Pondicherry, JIPMER was 13.5% [21]. Hospital records of the Burdwan Medical College (BMC) of West Bengal for the year 2009 shows, out of 1424 venomous snake bite cases treated there, 74 died (case fatality rate 5.19%) [6]. From Table 4, it is evident from the present study that the maximum number of snakebites were reported from Garbetta I, Chandrakona II and Debra. From the other blocks there has been no reported cases of snakebite deaths from hospital data.

Snake bite and death rate is always high in the rainy season [22]. In the present study the maximum(34%) number of snakebite was reported in the months of June-September. Earlier in several studies the incidence of snake bite was found in the months of June-September [23]. The agewise distribution of snakebite cases show that majority of snakebite affected cases were within the age of 21–45 years. Previous reports show maximum number of snakebite in the age group of 15–45 years [6]. However due to under reporting in majority of the cases the age of the cases were not mentioned. In the present study about 20% patients developed pyrogenic responses and anaphylactic response after antiserum administration and were treated with adrenaline. This finding is consistent with a double-blind placebo-controlled trial, conducted in Polonnaruwa, Sri Lanka, the same ASV was utilized and it was established that premedication with adrenaline significantly reduces the risk of acute adverse reactions to antivenom sera [24]. Earlier Theakston et al., [20] also found in 33% cases of anaphylactic responses 50 min after the initial ASV dose which is also corroborated in this study. There are several reports showing benefit of acetylcholinesterase inhibitors(AchEIs) in envenoming by kraits [25]. PLA2 s from snake venom neurotoxins produce complex effects on the pre-synaptic nerve terminal. These include entry into nerve terminals after binding to specific receptors on the pre-synaptic membrane, morphological changes such as nerve terminal bulging, changes in mitochondrial morphology and permeability, increase in cytosolic calcium levels, changes in expression and interactions of SNARE proteins, increased vesicle fusion and neurotransmitter release, and impaired vesicle recycling. Montecucco and colleagues have shown that the effects produced by four different snake venom PLA2 s (beta-bungarotoxin, taipoxin, notexin, and textilotoxin) were similar, suggesting a similar mechanism of action for pre-synaptic neurotoxins. Hydrolysis of the phospholipids of the pre-synaptic membrane and membrane destabilization by the products of hydrolysis are likely to be key drivers in this process [26], [27], [28]. The envenoming snake species is highly likely to influence the clinical presentation and outcome, but many studies have considered together bites from different snake species [29]. Such differences are perhaps unavoidable as confirming the identity of the envenoming snake is often difficult. Only a few studies have reported snake identification by detection of venom antigens [30], [31], [32].

## 5 Conclusion

Snakebite in *Midnapore* is a public health problem which is worsened by the unavailability of antivenom. The burden of snakebite in this district perhaps due to underreporting is difficult to estimate because the victims donot seek medical help from government dispensaries. Besides, differing environments, human activities, such as resettlement, sheepherding, fire wood-collecting, defecating, sugar-cane cutting, honey collecting, timber working, encroaches to differing extents on the serene life of snakes. Underreporting of snake bite occurrences have contributed to the variations in observed incidence. But, considering the heterogeneity of medical care and reporting and traditional cultural attitudes to snakes and snake bites, it seems likely that snake bite in Midnapore is widely underreported. For example, in some countries, up to 80% of snake bite victims seek treatment with traditional healers [3] rather than government dispensaries or hospitals. There is an urgent need for case documentation and reporting of the snakebite incidence and determinants in *Paschim Midnapore* district, West Bengal. In the present study we have attempted to report the hospital based official snakebite statistics (2012–2016) from 10 blocks of this district which actually reflects part of the snakebite scenario of the district. Further documentation of snakebite data from community based surveys and information from traditional healers can provide substantial information about the snakebite statistics of this district.
